# Use of late-night salivary cortisol to monitor response to medical treatment in Cushing’s disease

**DOI:** 10.1530/EJE-19-0695

**Published:** 2019-12-03

**Authors:** John Newell-Price, Rosario Pivonello, Antoine Tabarin, Maria Fleseriu, Przemysław Witek, Mônica R Gadelha, Stephan Petersenn, Libuse Tauchmanova, Shoba Ravichandran, Pritam Gupta, André Lacroix, Beverly M K Biller

**Affiliations:** 1 The Medical School, University of Sheffield, Sheffield, UK; 2 Università Federico II di Napoli, Naples, Italy; 3 Department of Endocrinology, CHU of Bordeaux, Bordeaux, France; 4 Northwest Pituitary Center, Oregon Health & Science University, Portland, Oregon, USA; 5 Department of Endocrinology, Military Institute of Medicine and Department of Internal Medicine, Endocrinology and Diabetes, Medical University of Warsaw, Warsaw, Poland; 6 Hospital Universitário Clementino Fraga Filho, Universidade Federal do Rio de Janeiro, Rio de Janeiro, Brazil; 7 ENDOC Center for Endocrine Tumors, Hamburg, Germany; 8 Novartis Pharma AG, Basel, Switzerland; 9 Novartis Pharmaceuticals Corporation, East Hanover, New Jersey, USA; 10 Novartis Healthcare Private Limited, Hyderabad, India; 11 Centre Hospitalier de l’Université de Montréal, Montreal, Canada; 12 Neuroendocrine Clinical Center, Massachusetts General Hospital, Boston, Massachusetts, USA

## Abstract

**Objective:**

Monitoring of patients with Cushing’s disease on cortisol-lowering drugs is usually performed with urinary free cortisol (UFC). Late-night salivary cortisol (LNSC) has an established role in screening for hypercortisolism and can help to detect the loss of cortisol circadian rhythm. Less evidence exists regarding the usefulness of LNSC in monitoring pharmacological response in Cushing’s disease.

**Design:**

Exploratory analysis evaluating LNSC during a Phase III study of long-acting pasireotide in Cushing’s disease (clinicaltrials.gov: NCT01374906).

**Methods:**

Mean LNSC (mLNSC) was calculated from two samples, collected on the same days as the first two of three 24-h urine samples (used to calculate mean UFC [mUFC]). Clinical signs of hypercortisolism were evaluated over time.

**Results:**

At baseline, 137 patients had evaluable mLNSC measurements; 91.2% had mLNSC exceeding the upper limit of normal (ULN; 3.2 nmol/L). Of patients with evaluable assessments at month 12 (*n* = 92), 17.4% had both mLNSC ≤ULN and mUFC ≤ULN; 22.8% had mLNSC ≤ULN, and 45.7% had mUFC ≤ULN. There was high variability in LNSC (intra-patient coefficient of variation (CV): 49.4%) and UFC (intra-patient CV: 39.2%). mLNSC levels decreased over 12 months of treatment and paralleled changes in mUFC. Moderate correlation was seen between mLNSC and mUFC (Spearman’s correlation: ρ = 0.50 [all time points pooled]). Greater improvements in systolic/diastolic blood pressure and weight were seen in patients with both mLNSC ≤ULN and mUFC ≤ULN.

**Conclusion:**

mUFC and mLNSC are complementary measurements for monitoring treatment response in Cushing’s disease, with better clinical outcomes seen for patients in whom both mUFC and mLNSC are controlled.

## Introduction

Cushing’s disease is a rare, serious and debilitating disorder of endogenous hypercortisolism, which is caused by an adrenocorticotropic hormone (ACTH)-secreting pituitary tumour (1). Prompt and effective treatment is required to reduce the considerable morbidity and mortality associated with Cushing’s disease (2). Transsphenoidal surgery is the first-line treatment for most patients (2). However, other treatment options, including medical therapy, are frequently required following surgical failure or disease recurrence, as well as for patients who are not candidates for surgery (3). Medical treatment options to control hypercortisolism, a key goal in managing patients with Cushing’s disease, include the second-generation somatostatin analogue pasireotide, adrenal steroidogenesis inhibitors and dopamine receptor agonists (3). Mifepristone, a glucocorticoid receptor antagonist, can also be used in patients with hyperglycaemia associated with Cushing’s syndrome, but it does not decrease cortisol production (3).

Assessment of 24-h urinary free cortisol (UFC) and late-night salivary cortisol (LNSC) levels has an established role in the initial screening for hypercortisolism and in the detection of disease recurrence after surgery (4). LNSC has also been used in combination with morning salivary cortisol levels to assess cortisol diurnal rhythm, which is frequently disrupted in patients with Cushing’s disease (5, 6). Despite this, there remains a lack of guidance on the most appropriate way to monitor disease activity during medical treatment. UFC is commonly assessed in clinical trials and during routine clinical practice to assess response to treatment. However, issues include difficulties obtaining a complete 24-h urine sample and high intra-patient variability on a day-to-day basis (7). In addition, UFC cannot provide information on circadian fluctuations of cortisol or midnight cortisol levels, which have been shown to play a role in the morbidity of dysregulated cortisol secretion (8, 9, 10, 11). Collection of late-night salivary samples is both simple and convenient for patients to carry out in their own homes, with samples being stable at room temperature for up to 2 weeks and easy to store (12, 13). Few studies have examined the role of LNSC in monitoring response to pharmacotherapy in patients with Cushing’s disease (6, 14).

The aim of the current exploratory analysis was to evaluate changes in LNSC during medical treatment with long-acting pasireotide during a large Phase III clinical trial in patients with Cushing’s disease and explore its relationship with UFC levels and clinical signs of hypercortisolism (15).

## Subjects and methods

### Patients and study design

Full details of the study design for this multicentre, 12-month, Phase III study have been described previously (15). In brief, adult patients with a confirmed diagnosis of persistent, recurrent or *de novo* (if not candidates for surgery) Cushing’s disease who had mean UFC (mUFC; calculated from three 24-h samples) of 1.5–5.0 times the upper limit of normal (ULN) at screening were enrolled.

Following screening and appropriate washout of medications used to treat Cushing’s disease, patients were randomized (1:1) to long-acting, i.m. pasireotide 10 mg or 30 mg every 4 weeks for 12 months. The dose could be increased (10 mg to 30 mg and 30 mg to 40 mg) at month 4 if mUFC >1.5× ULN and at month 7, 9 or 12 if mUFC >1.0× ULN. Dose reductions were permitted at any time for safety and tolerability.

The study was conducted in accordance with the Declaration of Helsinki, and an independent ethics committee or institutional review board for each site approved the study protocol. Patients provided written informed consent.

### Study objectives and assessments

A pre-specified exploratory objective of the study was to evaluate LNSC levels during treatment. Two LNSC samples per time point were collected using provided polypropylene tubes at 23:00 ± 1 h at baseline, during months 1–7 and at months 9 and 12. The two LNSC samples were collected on the same days as the first two of three 24-h UFC samples, which were collected within a 2-week period prior to the administration of the study drug. UFC samples and a single morning serum cortisol sample were collected at baseline and months 1–12. At each time point, mean LNSC (mLNSC) levels were calculated for each patient from the two samples (or a single LNSC value if one LNSC sample was missing), while mUFC was calculated from three samples (or two samples if one UFC sample was missing). For correlative analyses between single LNSC and UFC values, only the UFC samples collected on the same days as the LNSC samples were used. Patients were provided with a collection kit and an instruction sheet telling them how to collect and store the LNSC sample; patients collected the sample prior to brushing their teeth. The primary objective of the study, which has been reported previously (15), was to assess the efficacy of long-acting pasireotide as determined by the proportion of patients with mUFC ≤ULN at month 7. Changes in mUFC and serum cortisol levels during treatment were secondary objectives.

LNSC, UFC and morning serum cortisol levels were analysed at one of two central laboratories (Quintiles, Marietta, GA, USA, and Q^2^ Solutions [Beijing] Co Ltd, Beijing, China, depending on the location of the participating centre) by HPLC–tandem mass spectrometry (HPLC-MS/MS; Waters Corp, Milford, MA, USA). Reference ranges were established by measuring LNSC, UFC and morning serum cortisol levels in healthy male and female subjects (60 each for LNSC; 65 each for UFC and serum cortisol): LNSC, 0.2–3.2 nmol/L (7.4–116.0 ng/dL); UFC, 15.9–166.5 nmol/24 h (5.8–60.3 µg/24 h); and morning serum cortisol, 146.2–532.2 nmol/L (5.3–19.3 µg/dL). The intra- and inter-assay coefficients of variation (CVs) respectively were LNSC, 1.8–3.2% and 3.5–4.5%; UFC, 2.4–7.1% and 4.5–5.6%; and serum cortisol, 1.7–3.9% and 4.6–5.8%.

Changes in clinical signs of hypercortisolism and health-related quality of life (HRQoL; assessed using the CushingQoL questionnaire) were secondary objectives of the study. Blood pressure (supine), body weight, waist circumference and lipid profile were assessed at baseline and then monthly from months 1 to 12; CushingQoL score was assessed at baseline and months 2, 4, 7, 10 and 12.

### Statistical methods

All statistical analyses were performed using SAS 9.4 (SAS Institute, Inc, Cary, NC, USA). Data are shown as mean ([s.d.) or median (range), unless otherwise stated. Data from each randomized group were pooled for all analyses. For some analyses, patients were stratified according to mLNSC control status (controlled, ≤ULN; uncontrolled, >ULN) and/or mUFC control status (controlled, ≤ULN; partially controlled, >ULN and ≥50% reduction from baseline; uncontrolled, >ULN and <50% reduction from baseline), as defined in the study protocol. Change from baseline in mLNSC was initially calculated within each patient, followed by the overall mean change.

Correlations between individual LNSC and UFC values, mLNSC and mUFC were evaluated using the Spearman’s rank test. Correlations were also performed for (i) the lower and (ii) the higher of each patient’s two LNSC values at each time point with the UFC sample collected on the same day.

Only patients with both UFC and LNSC assessments within the same 24-h period at the specific time point were included. Correlation between mean change from baseline to month 12 in mLNSC and mean changes in clinical features of hypercortisolism was also evaluated using the Spearman’s rank test. Intra-patient CV was calculated between the two LNSC samples and between the two corresponding UFC samples. Intra-patient CVs for LNSC and UFC were calculated using the root-mean-square approach: the CV for each subject was identified, squared and the square root of the mean calculated. The Feltz and Miller asymptotic test was used to test the equality of CVs. Correlation was also calculated using the Pearson correlation coefficient.

Intra-patient CVs for LNSC and UFC, as well as mean mLNSC and mUFC levels, at baseline and change to month 12 were also assessed according to the patient age (<60 and ≥60 years), sex, diabetic status and hypertensive status. As serum cortisol was collected in the morning, correlative analyses with LNSC were not deemed relevant given the likely variability.

Mean changes and corresponding 95% confidence intervals (95% CI) in clinical signs of hypercortisolism and CushingQoL score from baseline to month 12 were stratified according to combined mLNSC and mUFC control at month 12.

## Results

### Study population

Overall, 137 of 150 (91.3%) patients who were enrolled in the study had an mLNSC assessment at baseline (Table 1). Most of these patients (81.0%) had persistent or recurrent disease after previous pituitary neurosurgery, and 41.6% had received previous medical therapy for Cushing’s disease. More than half of patients (56.2%) were classified as diabetic or pre-diabetic at study entry.

**Table 1:** Baseline characteristics for patients with baseline mLNSC assessment.

	All patients (*n* = 137)
Mean age, years (s.d.)	38.3 (12.9)
Female, *n* (%)	107 (78.1)
Cushing’s disease status, *n* (%)	
Persistent or recurrent	111 (81.0)
*De novo*	26 (19.0)
Previous medical therapy, *n* (%)	57 (41.6)
Mean mUFC (s.d.)	
nmol/24 h	478.7 (303.6)
× ULN	2.9 (1.8)
Mean mLNSC (s.d.)	
nmol/L	10.4 (8.2)
× ULN	3.3 (2.6)
Baseline diabetic status,* *n* (%)	
Diabetic	54 (39.4)
Pre-diabetic	23 (16.8)
Non-diabetic	60 (43.8)
Baseline hypertension status,^†^ *n* (%)	
Hypertensive	98 (71.5)
Pre-hypertensive	26 (19.0)
Normotensive	13 (9.5)

*Defined as follows: diabetic: receiving antidiabetic medication, or history of diabetes mellitus, or HbAlc (HbA_1c_) ≥6.5% or fasting plasma glucose (FPG) ≥126 mg/dL; pre-diabetic: not qualifying as diabetic and with FPG 100–126 mg/dL or HbA_1c_ 5.7–6.5%; non-diabetic: not qualifying as diabetic or pre-diabetic. ^†^Defined as follows: hypertensive: receiving antihypertensive medication, or systolic blood pressure (SBP) ≥140 mmHg or diastolic blood pressure (DBP) ≥90 mmHg; pre-hypertensive: SBP 120 to <140 mmHg or DBP 80 to <90 mmHg without antihypertensive medication; normotensive: not qualifying as hypertensive or pre-hypertensive (16).

### LNSC and UFC variability

**Figure 1:**
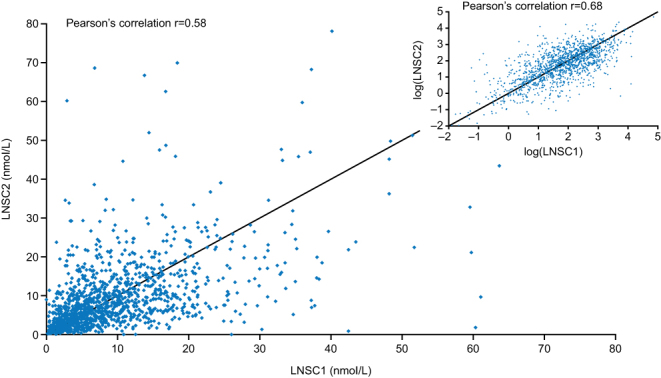
Scatter and log-scatter plot of LNSC values for paired samples (all sample collections). Inset shows scatter plot between pairs of LNSC values after log-transformation. LNSC1, first LNSC sample; LNSC2, second LNSC sample.

Most (97.1% [*n* = 1353/1394]) pairs of LNSC samples were collected on consecutive days (range: 1.0–10.0 days). For patients with LNSC assessments, the group mean (s.d.) values were similar for samples 1 and 2 both at baseline (10.5 [8.2] vs 10.2 [10.1] nmol/L) and when all time points were pooled (9.1 [9.2] vs 8.9 [9.5] nmol/L). However, the intra-patient CV for the two samples was 45.2% at baseline, 45.8% at month 12 and 49.4% when all time points were pooled (Pearson’s correlation, *r* = 0.58). The analysis of individual patients showed a high degree of variability between paired LNSC samples in some patients (Fig. 1). The intra-patient CV for LNSC was higher (at baseline and all pooled time points) in patients who were normotensive at baseline than in patients with pre-hypertension or hypertension. No differences in LNSC variability were seen when patients were stratified by baseline mLNSC levels, diabetic status, age (<60 and ≥60 years) and sex (Table 2).

**Table 2:** Intra-patient CV (%) for LNSC at baseline and over all time points.

	*n*	Baseline	All time points
Baseline mLNSC × ULN			
≤1.5	73	45.6	51.5
1.5–2.0	28	46.1	44.9
2.5–5.0	17	42.0	49.0
5.0–10.0	3	44.2	53.2
Age, years			
<60	113	44.9	49.7
≥60	8	48.5	45.9
Sex			
Female	94	43.6	49.4
Male	27	50.4	49.5
Baseline diabetic status			
Diabetic	48	41.0	48.2
Pre-diabetic	17	44.4	49.3
Non-diabetic	56	48.7	50.5
Baseline hypertensive status			
Hypertensive	84	42.6	48.4
Pre-hypertensive	24	35.9	48.4
Normotensive	13	70.0	57.9

The two LNSC samples were taken within the same 24-h period as the first two of three UFC samples.

The intra-patient CV for the two UFC samples was 34.0% at baseline, 40.9% at month 12 and 39.2% when all time points were pooled; a high degree of variability between UFC samples was seen in some patients (Supplementary Fig. 1, see section on supplementary materials given at the end of this article). At baseline, intra-patient variability for UFC decreased as baseline mUFC increased and was higher in males versus females and in patients who were normotensive versus those with pre-hypertension or hypertension (Supplementary Table 1).

### Correlation between LNSC and UFC

At baseline, there was weak correlation between single LNSC and UFC values from samples that were collected on the same day (Spearman’s rank, ρ = 0.25) and for corresponding mLNSC and mUFC values (ρ = 0.24). The strength of correlation was greater, following 12 months of long-acting pasireotide treatment for both single (ρ = 0.43) and mean (ρ = 0.48) values (Table 3). Similar correlations were seen when data from all time points were pooled (Table 3).

**Table 3:** Pairwise correlation between single and mean LNSC and UFC values.

	Number of samples	Spearman’s rank correlation (ρ)	*P* value
Baseline			
LNSC vs UFC	252	0.25	<0.001
mLNSC vs mUFC	137	0.24	0.006
Lower LNSC value vs UFC	130	0.28	0.001
Higher LNSC value vs UFC	128	0.22	0.014
Month 12			
LNSC vs UFC	180	0.43	<0.001
mLNSC vs mUFC	92	0.48	<0.001
Lower LNSC value vs UFC	91	0.43	<0.001
Higher LNSC value vs UFC	92	0.39	<0.001
All months			
LNSC vs UFC	2908	0.46	<0.001
mLNSC vs mUFC	1489	0.50	<0.001
Lower LNSC value vs UFC	1472	0.46	<0.001
Higher LNSC value vs UFC	1469	0.47	<0.001

LNSC and UFC samples were collected within the same 24-h period.

Similar correlations were also seen for the lower and the higher of the two LNSC values, with the UFC value from the sample collected on the same day (month 12: ρ = 0.43 vs ρ = 0.39) and when all time points were pooled (ρ = 0.46 vs ρ = 0.47) (Table 3).

### Effect of long-acting pasireotide on mLNSC and mUFC levels

At baseline, 91.2% (*n* = 125/137) of patients with an evaluable assessment had mLNSC >ULN (>3.2 nmol/L). Of the 12 patients who had mLNSC ≤ULN at baseline, mUFC was ≤ULN in two patients and >ULN in 10 patients (all patients met the eligibility criterion of mUFC ≥1.5× ULN at screening visit). Mean (s.d.) mUFC for the 12 patients with mLNSC ≤ULN was 300.1 (157.0) nmol/24 h (1.8 [1.1] × ULN). Mean (SD) mLNSC level at baseline was 10.4 (8.2) nmol/L (3.3 [2.6] × ULN), with no apparent differences when patients were stratified by sex, diabetic status or hypertensive status (Supplementary Table 2). mLNSC was higher in patients aged ≥60 years versus <60 years (15.9 [14.8] vs 10.0 [7.2] nmol/L).

**Figure 2:**
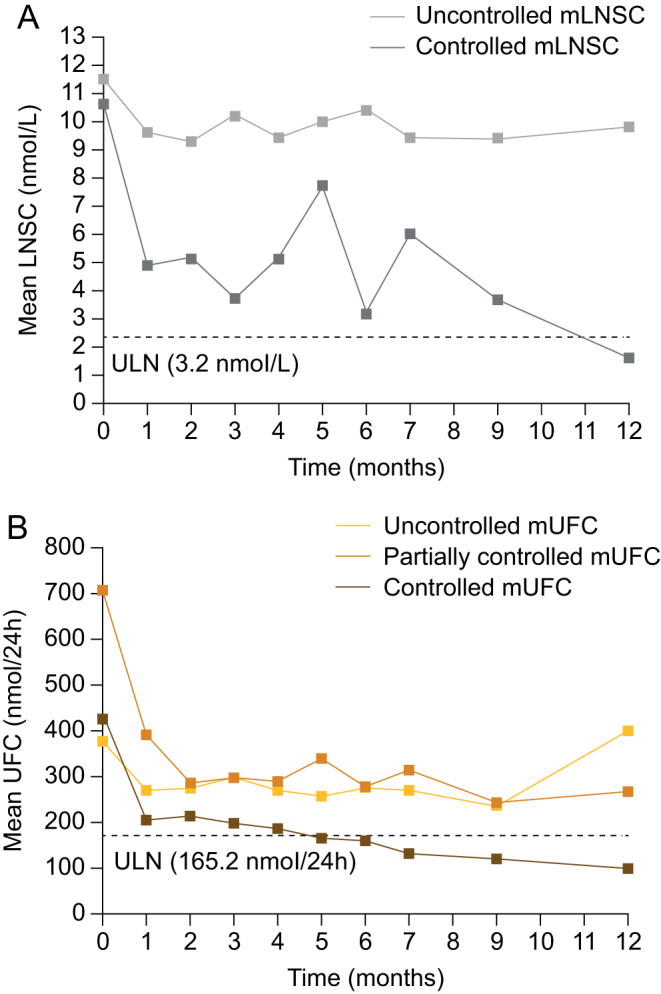
(A) mLNSC and (B) mUFC levels at baseline and during long-acting pasireotide treatment according to mLNSC and mUFC response status respectively at month 12. mLNSC and mUFC control status was defined by study protocol definitions. Controlled mLNSC: mLNSC ≤ULN; uncontrolled mLNSC: mLNSC >ULN. Controlled mUFC: mUFC ≤ULN; partially controlled mUFC: mUFC >ULN but ≥50% reduction in mUFC from baseline; uncontrolled mUFC: mUFC >ULN and <50% reduction from baseline.

Mean mLNSC levels decreased during long-acting pasireotide treatment. Mean (95% CI) change in mLNSC from baseline to month 7 was −1.6 nmol/L (−3.7, 0.5; *n* = 108) and to month 12 was −3.3 nmol/L (−5.6, −1.0; *n* = 86). When stratified by mLNSC response status at month 12, the mean change from baseline in mLNSC at month 12 was −9.0 nmol/L (−16.5, −1.4; *n* = 19) in patients with controlled mLNSC and −1.7 nmol/L (−3.7, 0.4; *n* = 67) in patients with uncontrolled mLNSC (Fig. 2A). mLNSC was reduced at month 12 compared with baseline for patients with controlled mUFC (−6.9 [−11.4, −2.4]; *n* = 37) but not partially controlled or uncontrolled mUFC at month 12 (Table 4). Reductions in mLNSC levels were numerically higher in females, patients aged ≥60 years and patients who were normotensive at baseline (Table 4). It is, however, important to note that the unequal distribution of patients between subgroups and the small number of patients who were normotensive (*n* = 9), ≥60 years old (*n* = 6) and male (*n* = 22) hinder the interpretation of any differences between subgroups.

**Table 4:** Mean (95% CI) change from baseline in mLNSC and mUFC at month 12.

	mLNSC (nmol/L)		mUFC (nmol/24 h)	
	*n*	Change from baseline at month 12	*n*	Change from baseline at month 12
All	86	−3.3 (−5.6, −1.0)	104	−222.4 (−288.5, −156.2)
mUFC control at month 12				
Controlled	37	−6.9 (−11.4, −2.4)	45	−326.9 (−430.8, −223.1)
Partially controlled	18	0.8 (−3.1, 4.6)	21	−440.9 (−532.9, −348.9)
Uncontrolled	31	−1.3 (−3.7, 1.2)	38	22.3 (−55.7, 100.2)
mLNSC control at month 12*				
Controlled	19	−9.0 (−16.5, −1.4)	21	−330.2 (−557.7, −102.7)
Uncontrolled	67	−1.7 (−3.7, 0.4)	71	−213.8 (−273.5, −154.2)
Diabetic status at baseline				
Diabetic	33	−3.5 (−8.1, 1.1)	41	−235.7 (−337.2, −134.3)
Pre-diabetic	13	−2.6 (−6.9, 1.7)	17	−120.0 (−298.1, 58.0)
Non-diabetic	40	−3.3 (−6.5, −0.1)	46	−248.2 (−353.5, −143.0)
Hypertension status at baseline				
Hypertensive	62	−3.0 (−5.9, −0.2)	75	−185.4 (−256.2, −114.6)
Pre-hypertensive	15	−2.7 (−4.6, −0.8)	18	−308.4 (−492.2, −124.6)
Normotensive	9	−5.8 (−17.8, 6.2)	11	−333.6 (−649.7, −17.6)
Age				
<60 years	80	−2.5 (−4.5, −0.4)	97	−218.4 (−283.1, −153.6)
≥60 years	6	−14.0 (−36.4, 8.4)	7	−277.4 (−809.5, 254.6)
Sex				
Male	22	−1.7 (−5.2, 1.9)	24	−150.4 (−242.4, −58.4)
Female	64	−3.8 (−6.7, −0.9)	80	−243.9 (−325.7, −162.2)

*92 of the 104 patients who had mUFC assessments at baseline and month 12 (for calculation of mean change) also had mLNSC assessments at each of these time points.

Mean (s.d.) mUFC level at baseline was 470.0 (296.1) nmol/24 h (2.8 [1.8] × ULN). There were no clear differences in baseline mean mUFC when patients were stratified according to sex or diabetic or hypertensive status at baseline (Supplementary Table 2), although mean mUFC was higher in patients aged ≥60 years than in those aged <60 years (588.9 [455.4] vs 460.6 [280.0] nmol/24 h).

Mean mUFC levels decreased during 12 months of treatment, overall, and for patients with controlled and partially controlled mUFC at month 12 (Fig. 2B and Table 4). In patients with uncontrolled mUFC, mUFC decreased over time to month 9 and then increased at month 12 (Fig. 2). Patients who were hypertensive at baseline had less improvement in mean mUFC than those who were pre-hypertensive or normotensive. Mean changes in mUFC from baseline to month 12 were similar when patients were stratified according to diabetic status, age or sex.

### Normalization of mLNSC and mUFC during treatment with long-acting pasireotide

**Figure 3:**
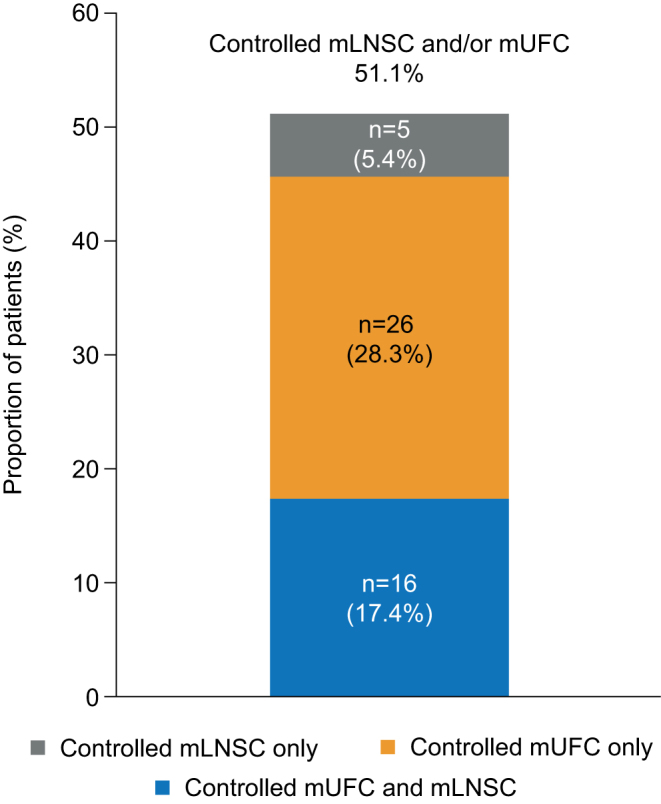
Proportion of patients with controlled mLNSC and/or mUFC at month 12 (*n* = 92). Results are shown only for patients with evaluable mLNSC and mUFC. Controlled mLNSC: mLNSC ≤ULN; controlled mUFC: mUFC ≤ULN.

Of the 113 patients with both mLNSC and mUFC assessments at month 7, controlled (≤ULN) mLNSC and mUFC levels were seen in 25 (22.1%) and 56 (49.6%) patients respectively. Twenty (17.7%) patients had both controlled mLNSC and mUFC, 36 (31.9%) patients had elevated mLNSC in the presence of controlled mUFC and five (4.4%) patients had normal mLNSC but elevated mUFC. Of 92 patients with both an mLNSC and mUFC assessment at month 12, 21 (22.8%) had controlled mLNSC and 42 (45.7%) had controlled mUFC; 16 (17.4%) patients had both controlled mLNSC and mUFC, 26 (28.3%) patients had elevated mLNSC in the presence of controlled mUFC, while five (5.4%) had normal mLNSC but elevated mUFC (Fig. 3).

Of the 21 patients who had controlled mLNSC at month 12, both LNSC values were ≤ULN in 16 (76.2%). Of patients with controlled mUFC at month 12, 41/42 (97.6%) had at least two samples in the normal range. Four of 26 (15.4%) patients who had elevated mLNSC in the presence of normal mUFC at month 12 had one LNSC sample ≤ULN. Of the five patients who had normal mLNSC but elevated mUFC at month 12, both LNSC samples were ≤ULN in four (80.0%). One patient with controlled mLNSC had a morning serum cortisol level below the lower limit of normal.

### Effect of mLNSC and/or mUFC control on clinical signs of hypercortisolism and HRQoL

**Figure 4:**
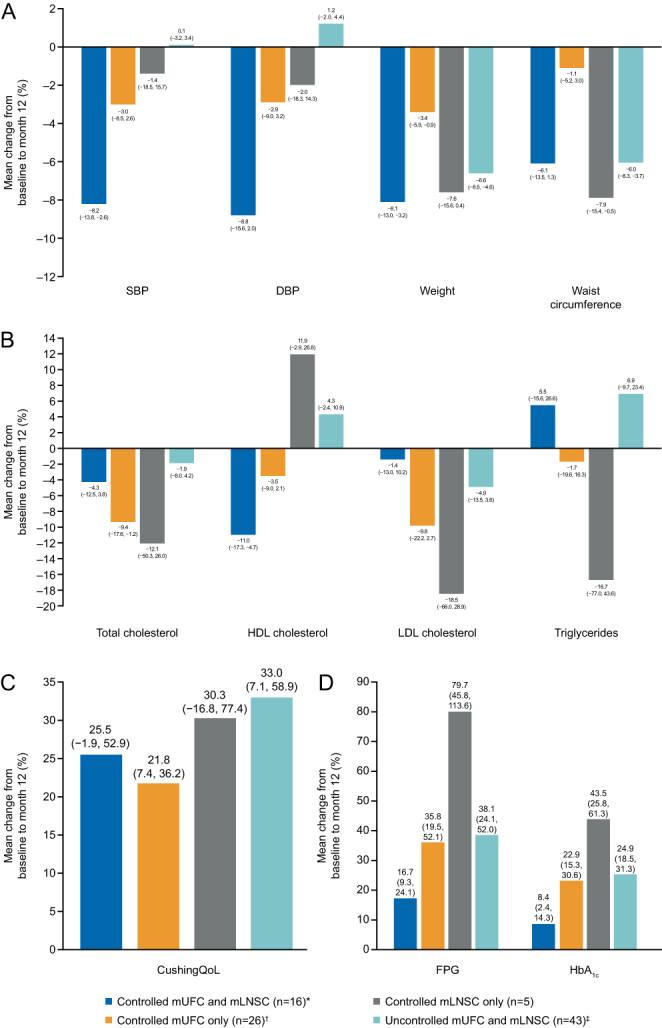
Mean (95% CI) percentage change from baseline to month 12 in (A) clinical signs, (B) lipid profile, (C) CushingQoL score and (D) glycaemic parameters by mUFC and mLNSC control status. Controlled mLNSC: mLNSC ≤ULN; controlled mUFC: mUFC ≤ULN. *n = 13 for waist circumference and *n* = 15 for CushingQoL; ^†^*n* = 25 for waist circumference and lipids; ^‡^*n* = 44 for weight, *n* = 41 for waist circumference and lipids and *n* = 42 for CushingQoL. HDL, high-density lipoprotein; LDL, low-density lipoprotein.

Improvements in clinical signs and HRQoL during 12 months of treatment with long-acting pasireotide have already been reported for the overall study population (15). When stratifying patients according to mLNSC and mUFC response at month 12, improvements in systolic blood pressure (SBP) and diastolic blood pressure (DBP) were greatest in patients with both mLNSC ≤ULN and mUFC ≤ULN (mean [95% CI] percentage change: −8.2% [−13.8, −2.6] and −8.8% [−15.6, −2.0]). SBP and DBP were numerically lower at month 12 than at baseline in patients with mLNSC ≤ULN but mUFC >ULN, and vice versa (Fig. 4A). At month 12, reductions in mean weight and waist circumference were reported across all mLNSC and mUFC response groups (Fig. 4A). Decreases in total cholesterol and LDL cholesterol were seen in all mLNSC/mUFC response groups (Fig. 4B). CushingQoL score was numerically higher at month 12 than at baseline in all mUFC and mLNSC response groups (Fig. 4C).

Fasting plasma glucose (FPG) and glycated haemoglobin (HbA_1c_) increased from baseline to month 12 in all mLNSC/mUFC response groups (Fig. 4D). The smallest numerical increases in FPG and HbA_1c_ were seen in patients with both mLNSC ≤ULN and mUFC ≤ULN (mean [95% CI] percentage change: FPG, 16.7 [9.3, 24.1]; HbA_1c_, 8.4 [2.4, 14.3]).

## Discussion

While LNSC has an established role in the diagnosis of Cushing’s disease and prediction of recurrence risk after surgery, its usefulness as a biomarker of medical treatment response is not yet known (5, 6, 16, 17). The current analysis, which included a large subset of patients enrolled in a Phase III study, provides further evidence supporting a role for LNSC in monitoring patients with active Cushing’s disease who are receiving medical treatment. An overall decrease in mean mLNSC levels was seen over 12 months of treatment with long-acting pasireotide, which mirrored changes in mUFC. At month 12, 23% (*n* = 21/92) of patients had normalized mLNSC and 46% of patients achieved normalized mUFC (*n* = 42/92). Improvements in systolic and diastolic blood pressure were greatest in patients who achieved normalization of both mLNSC and mUFC.

UFC is commonly used in clinical studies and routine practice to monitor response to medical therapy in patients with Cushing’s disease. However, there are important limitations to the use of UFC, which include the need for a patient to collect a complete 24-h urine sample (18, 19), as well as high intra-patient variability in daily 24-h UFC measurements (7). Consistent with findings from a previous exploratory analysis of single LNSC samples from a large Phase III study of s.c. pasireotide in Cushing’s disease (14), we found a moderate correlation between individual and mean UFC and LNSC values, the samples for which were collected in the same 24-h period. In the current analysis, 32% and 28% of patients had an elevated mLNSC level at months 7 and 12, respectively, in the presence of normalized mUFC. These findings are likely to be of clinical importance given that improvements in systolic and diastolic blood pressure were greater in patients who had normal mLNSC and mUFC levels compared with patients who had discordant mUFC and mLNSC. Similar results have also been reported during cabergoline and ketoconazole combination therapy, with greater improvements in blood pressure seen in those patients who had both UFC and LNSC levels normalized during treatment compared with those who had only UFC normalized (16). Taken together, these findings suggest that simultaneous control of both daily cortisol secretion (measured by UFC) and late-night cortisol levels (at the circadian nadir) is likely to be an important treatment goal for patients with Cushing’s disease, and that care should be taken when assessing the efficacy of any given treatment option based on the control of mUFC or mLNSC alone. Future clinical trials for Cushing’s disease could consider a composite endpoint of both controlled UFC and late-night cortisol levels as a key treatment objective.

Hyperglycaemia is a well-characterized side effect of treatment with pasireotide (20). In this analysis, FPG and HbA_1c_ levels increased from baseline to month 12 in all mLNSC/mUFC response groups. The smallest numerical increases in these glycaemia parameters were seen in patients who achieved normalization of both mLNSC and mUFC. As use of antidiabetic medication was permitted during this study, additional investigations are required to determine whether the correction of hypercortisolism can attenuate increases in blood glucose associated with pasireotide treatment. A separate study that was specifically designed to investigate the optimal management of pasireotide-associated hyperglycaemia has recently been completed (clinicalTrials.gov: NCT02060383) and will be reported elsewhere.

In our study, elevated mLNSC in patients with normal mUFC may have resulted from suboptimal recovery of normal circadian cortisol rhythm – characterized by an early-morning cortisol peak and a gradual decrease during the day to low midnight levels (3) – despite an overall reduction in daily cortisol secretion. Indeed, dysregulation of cortisol circadian rhythm has been shown to persist in some patients during medical therapy even after normalization of mUFC (6, 14, 16). However, additional salivary cortisol assessments within a single day, including early in the morning and late at night, may be required to confirm the effects of medical therapy on circadian rhythm of cortisol secretion (6), which was not planned in the present study.

Emerging evidence has suggested that salivary cortisone may provide an improved measure of serum cortisol levels over salivary cortisol. Salivary cortisone is rapidly converted from serum free cortisol in the salivary gland, measurable at low serum cortisol levels and unaffected by oral hydrocortisone administration (21, 22). A recent study has demonstrated that three 8-hourly salivary cortisone samples may be sufficient to estimate overall cortisol exposure in healthy and patient populations. Confirmatory studies are required to assess the role of salivary cortisone in monitoring of patients with Cushing’s disease (23).

While higher salivary cortisol concentrations have previously been shown in untreated older, male patients with hypertension and diabetes, as well as in diabetic patients post-surgery (24, 25), this was confirmed only for older patients in our study. However, it is important to note that the small patient numbers in some subgroups hinder accurate interpretation. Additional studies are needed to determine the exact factors that influence mLNSC levels, with the aim of identifying whether age-, sex- and/or comorbidity-specific reference ranges are required.

Considerable day-to-day intra-patient variability has been described for both LNSC and UFC (7, 26, 27, 28). In our study, the intra-patient CV across two LNSC samples, which were almost always collected on consecutive days, was approximately 50% both at baseline and during treatment with long-acting pasireotide. The intra-patient CV was slightly lower (~40%) for the two UFC samples that were collected on the same day as the LNSC samples. Baseline mLNSC levels did not appear to influence LNSC variability, whereas intra-patient variability in UFC decreased as baseline mUFC levels increased. These findings differ from results of the previous exploratory analysis from the Phase III study of s.c. pasireotide, in which higher baseline mUFC levels were associated with increasing variability in UFC (7). These differences may be explained by the fact that, unlike in the study of s.c. pasireotide, patients with extreme elevations in mUFC were largely excluded from our study (eligibility criterion for our study was screening mUFC 1.5–5.0 × ULN vs ≥1.5 × ULN in the study of s.c. pasireotide). In the current study, patients were provided with an instruction sheet detailing how and when to collect their LNSC and UFC samples, which were assessed at a central laboratory by HPLC-MS/MS. As such, an even greater degree of variability in LNSC and UFC levels may be expected in routine clinical practice, especially if these levels are determined using an immunoassay method, which has lower accuracy than HPLC-MS/MS, or if patients are not given sufficient guidance on best practice for sample collection. It is important that clinicians are aware of the significant intra-patient variability in LNSC and UFC samples, as multiple samples are likely required to accurately interpret a patient’s response to medical treatment (29). The requirement for multiple LNSC samples is not likely to represent a burden for patients given that they are simple and convenient to collect in an outpatient setting, as well as being stable and easy to store (12).

This study had certain limitations, such as the exploratory nature of the analyses. Indeed, a main limitation of the analysis is that dose adjustments were made during the Phase III study based on mUFC levels and taking into consideration safety and tolerability but not considering LNSC levels. In future studies, the use of both mUFC and mLNSC assessments to guide dose optimization decisions would provide additional information on the effects of normalizing both biomarkers on improving clinical signs and HRQoL in patients with Cushing’s disease. In addition, the assessment of serum cortisol using a single, morning blood sample at each time point did not allow for an accurate analysis of its role in monitoring medical treatment responses in this study, as only a single serum cortisol sample was collected at each time point. Another limitation is that some subgroups had small sample sizes, limiting interpretation of the data. Finally, salivary cortisol levels can also be influenced by a range of extrinsic factors such as sleeping patterns, smoking and oral hygiene, but these were not systematically recorded as part of this study.

LNSC assessment is simple and convenient for patients with Cushing’s disease. LNSC demonstrates a high (~50%) degree of intra-patient variability on a day-to-day basis, similar to that seen for UFC. Determination of both mLNSC and mUFC can provide a more comprehensive assessment of medical treatment response in patients with Cushing’s disease. The normalization of both mLNSC and mUFC is likely to be of clinical importance given that patients who achieved normal mLNSC and mUFC levels showed the greatest clinical improvements.

## Supplementary Material

Supplementary Table 1. Intra-patient coefficient of variation (%) for UFC at baseline and over all time points

Supplementary Table 2. Mean (standard deviation) LNSC and UFC baseline levels by baseline characteristics and mUFC control at month 12

Supplementary Figure 1. Log-scale scatter plot of UFC values for paired samples collected at each time point (all sample collections)

## Declaration of interest

J N P has received research and consultancy fees (paid to the University of Sheffield) from Novartis, Ipsen, HRA Pharma and ONO Pharma. R P has received grants and personal fees from Novartis, Pfizer, HRA Pharma, Viropharma, Shire and Ipsen, personal fees from Ferring and Italfarmaco, and grants from Corcept Therapeutics, Cortendo AB and IBSA. A T has received research support and scientific consultancy fees from Novartis and HRA Pharma. M F has received research support (paid to Oregon Health & Science University) from Novartis, Millendo Therapeutics and Strongbridge Biopharma and scientific consultancy fees from Novartis and Strongbridge Biopharma. P W has received travel grants from Novartis and Ipsen Poland and consulting fees from Ipsen, Novartis and Pfizer. P W is the principal investigator in clinical trials sponsored by Novartis, Ipsen, Novo Nordisk and Strongbridge Biopharma. M G has been the principal investigator in clinical trials for Novartis and Ipsen and received consultancy fees from Novartis and speaker fees from Novartis, Ipsen and Pfizer. S P has served as a lecturer for Ipsen, Novartis and Pfizer and as an advisory board member for Ipsen and Novartis. L T, S R and P G are employees of Novartis. A L has received funding as an investigator or consultant from Novartis, Cortendo, Strongbridge Biopharma and GLWL Research, Inc. B M K B has been the principal investigator for research grants (paid to Massachusetts General Hospital) from Novartis, Millendo and Strongbridge Biopharma and has received occasional consulting honoraria from Novartis and Strongbridge Biopharma. André Lacroix is on the editorial board of EJE. André Lacroix was not involved in the review or editorial process for this paper, on which he/she is listed as an author.

## Funding

This study was sponsored by Novartis Pharma AG. Financial support for medical editorial assistance was provided by Novartis Pharmaceuticals Corporation.

## Author contribution statement

The study was designed by the academic investigators and the sponsor, Novartis Pharma AG. Data were collected by the investigators using Novartis’ data management systems and analysed by Novartis’ statistical team. The investigators enrolled patients in the study. All the authors contributed to data interpretation and writing, reviewing and amending of the manuscript; the first draft was prepared by a medical writer funded by Novartis Pharmaceuticals Corporation. All the authors made the decision to submit the manuscript for publication and vouch for the accuracy and completeness of the data.
