# Supplementary material for: Use of late-night salivary cortisol to monitor response to medical treatment in Cushing’s disease
Source: Eur J Endocrinol. 2019 Dec 3;182(2):207–17. doi: 10.1530/EJE-19-0695 (PMC7003692; doi:10.1530/EJE-19-0695)
Supplement: Supplementary Table 1. Intra-patient coefficient of variation (%) for UFC at baseline and over all time points [file supplementary_table_1.pdf]

Supplementary Table 1. Intra-patient coefficient of variation (%) for UFC at baseline and over all time points

	n	Baseline	All time points
Baseline mUFC, x ULN			
≤1.5	28	43.6	45.1
1.5–2.0	52	34.3	37.7
2.5–5.0	55	29.9	35.5
5.0–10.0	14	26.2	43.3
>10.0	1	13.8	55.3
Age, years			
<60	139	33.5	39.5
≥60	11	39.8	35.1
Sex			
Female	118	30.9	38.6
Male	32	43.8	40.9
Baseline diabetic status			
Diabetic	60	35.7	36.6
Pre-diabetic	24	38.1	39.7
Non-diabetic	66	30.7	41.1
Baseline hypertensive status			
Hypertensive	108	33.1	37.1
Pre-hypertensive	28	30.2	41.5
Normotensive	14	46.2	48.3

The two UFC samples were taken within the same 24-h period as the LNSC samples. LNSC, late-night salivary cortisol; mUFC, mean urinary free cortisol; ULN, upper limit of normal
