# Supplementary material for: Use of late-night salivary cortisol to monitor response to medical treatment in Cushing’s disease
Source: Eur J Endocrinol. 2019 Dec 3;182(2):207–17. doi: 10.1530/EJE-19-0695 (PMC7003692; doi:10.1530/EJE-19-0695)
Supplement: Supplementary Table 2. Mean (standard deviation) LNSC and UFC baseline levels by baseline characteristics and mUFC control at month 12 [file supplementary_table_2.pdf]

Supplementary Table 2. Mean (standard deviation) LNSC and UFC baseline levels by baseline characteristics and mUFC control at month 12

	n	mLNSC (nmol/L)	n	mUFC (nmol/24h)
All	137	10.4 (8.2)	150	470.0 (296.1)
Diabetic status at baseline				
Diabetic	54	11.6 (8.9)	60	455.2 (282.4)
Pre-diabetic	23	8.9 (6.3)	24	460.9 (273.9)
Non-diabetic	60	10.0 (8.1)	66	486.7 (318.6)
Hypertension status at baseline				
Hypertension	98	11.3 (8.3)	108	463.9 (277.0)
Pre-hypertension	26	7.4 (3.9)	28	486.1 (325.2)
Normotension	13	10.3 (11.9)	14	484.7 (390.6)
Age				
<60 years	126	10.0 (7.2)	139	460.6 (280.0)
≥60 years	11	15.9 (14.8)	11	588.9 (455.4)
Sex				
Male	30	10.1 (6.3)	32	409.3 (208.6)
Female	107	10.5 (8.6)	118	486.4 (314.4)

mLNSC, mean late-night salivary cortisol
