# Supplementary material for: Use of late-night salivary cortisol to monitor response to medical treatment in Cushing’s disease
Source: Eur J Endocrinol. 2019 Dec 3;182(2):207–17. doi: 10.1530/EJE-19-0695 (PMC7003692; doi:10.1530/EJE-19-0695)
Supplement: Supplementary Figure 1. Log-scale scatter plot of UFC values for paired samples collected at each time point (all sample collections) [file supplementary_figure_1.pdf]

Supplementary Figure 1. Log-scale scatter plot of UFC values for paired samples collected at each time point (all sample collections)

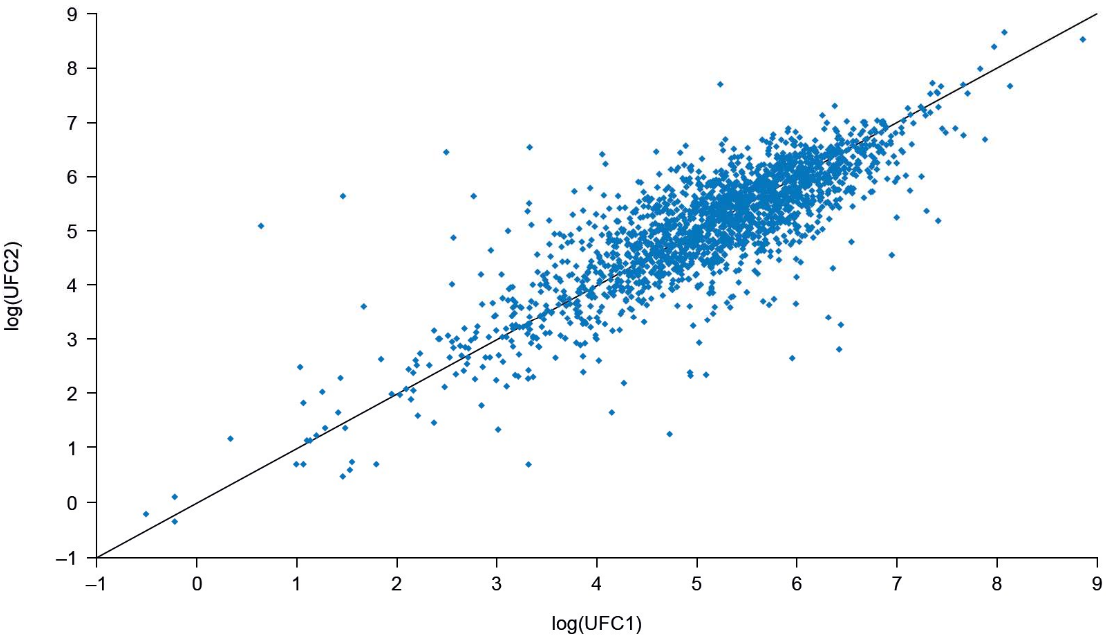

UFC1, first UFC sample; UFC2, second UFC sample
